# *In Vivo* Analysis of *Aicda* Gene Regulation: A Critical Balance between Upstream Enhancers and Intronic Silencers Governs Appropriate Expression

**DOI:** 10.1371/journal.pone.0061433

**Published:** 2013-04-16

**Authors:** Le Thi Huong, Maki Kobayashi, Mikiyo Nakata, Go Shioi, Hitoshi Miyachi, Tasuku Honjo, Hitoshi Nagaoka

**Affiliations:** 1 Department of Immunology and Genomic Medicine, Graduate School of Medicine, Kyoto University, Kyoto, Japan; 2 Department of Molecular Pathobiochemistry, Gifu University Graduate School of Medicine, Gifu, Japan; 3 Laboratory for Animal Resources and Genetic Engineering, RIKEN Center for Developmental Biology, Kobe, Japan; 4 Reproductive Engineering Team, Institute of Virus Research, Kyoto University, Kyoto, Japan; Michigan State University, United States of America

## Abstract

The *Aicda* gene encodes activation-induced cytidine deaminase (AID). *Aicda* is strongly transcribed in activated B cells to diversify immunoglobulin genes, but expressed at low levels in various other cells in response to physiological or pathological stimuli. AID’s mutagenic nature has been shown to be involved in tumor development. Here, we used a transgenic strategy with bacterial artificial chromosomes (BACs) to examine the *in vivo* functions of *Aicda* regulatory elements, which cluster in two regions: in the first intron (region 2), and approximately 8-kb upstream of the transcription start site (region 4). Deleting either of these regions completely abolished the expression of *Aicda*-BAC reporters, demonstrating these elements’ critical roles. Furthermore, we found that selectively deleting two C/EBP-binding sites in region 4 inactivated the enhancer activity of the region despite the presence of intact NF-κB-, STAT6- and Smad-binding sites. On the other hand, selectively deleting E2F- and c-Myb-binding sites in region 2 increased the frequency of germinal-center B cells in which the *Aicda* promoter was active, indicating that E2F and c-Myb act as silencers *in vivo*. Interestingly, the silencer deletion did not cause ectopic activation of the *Aicda* promoter, indicating that *Aicda* activation requires enhancer-specific stimulation. In summary, precise regulation of the *Aicda* promoter appears to depend on a coordinated balance of activities between enhancer and silencer elements.

## Introduction

Activation-induced cytidine deaminase (AID), which is encoded by *Aicda*, is central to antigen-induced immunoglobulin (Ig) diversification, that is, somatic hypermutation (SHM) and class-switch recombination (CSR) in activated B cells [1], [2]. AID triggers the induction of nicking cleavage at the *Ig* loci [3], [4]. Although the target specificity of AID is limited to *Ig* genes, in which SHM and CSR take place, AID can target many other genes, including proto-oncogenes, although with much lower frequencies [5]–[9]. It has been suggested that this type of non-physiological, off-target attack by AID is involved in tumorigenesis of not only B cells, but also other cell lineages [10]. Supporting this idea, artificially overexpressing AID in transgenic mice causes tumors in non-B cells such as T lymphoma, lung tumor, and hepatoma [11], [12]. Moreover, *Aicda* knockout drastically delays tumor development in animal models of plasmacytoma, which is associated with suppressed *Ig-Myc* translocation [13], [14]. AID is thus suspected to be an endogenous mutagenic enzyme.

Given this self-mutating activity of AID, one would expect *Aicda* to be regulated very strictly to avoid any leaky expression. Indeed, several reports have shown that strong AID expression is virtually restricted to germinal-center B cells *in vivo* [15]–[17]. However, there is growing evidence that cells other than activated B cells can express AID when exposed to strong stimulation. In particular, AID expression is induced in gastric epithelial cells by *Helicobacter pylori* infection, providing insight into the mechanisms of pathogen-associated tumorigenesis–namely, that AID may act as a mutagen in inflammation-associated tumor development [18]. Other tumorigenic pathogens, including the EB, hepatitis C, and HTLV-1 viruses, are reported to induce or enhance AID expression [19]–[22]. The NF-κB pathway appears to be involved in AID’s induction in *H. pylori*-infected gastric epithelial cells, suggesting that the inflammatory signals associated with infection by this pathogen may be involved in inducing AID [18].

Under physiological conditions, AID is also expressed in immature B cells and in some activated T cells at a level one to two orders of magnitude lower than that in germinal-center B cells; the role of such low amounts of AID is unclear [16], [17], [23], [24]. *Aicda* is transiently expressed in a fraction of activated T cells, including a subset of T cells that produces IL-10, suggesting that *Aicda*-inductive stimuli correlate with the development of this T-cell subset [17]. In the case of immature B cells, AID may assist in removing B cells carrying self-reactive Igs on their surface [25], [26]. These findings suggest that marginal levels of AID might have physiological roles other than Ig diversification, although the precise nature of those roles is largely unclear. Taken together, AID expression differs according to cell lineages and conditions, and these differences might account for the variety of physiological and pathological cellular responses elicited by this molecule.

Genome sequencing revealed four regions in and around *Aicda* that are well-conserved between humans and mice (regions 1, 2, 3, and 4) [16], [27], [28]. The downstream region (region 3) was shown to be important using *Aicda* bacterial-artificial-chromosome (BAC) constructs, although *in vitro* reporter assays have not clarified its regulatory function [16], [28]. Several *Aicda*-regulation studies have shown that the *Aicda* promoter (region 1) is not particularly specific to B cells, and promotes transcription in various types of cells [27], [28]. A HoxC4-binding site located in the promoter region is reported to induce *Aicda* transcription by increased HoxC4 levels [29], [30]. *Aicda* also contains major regulatory elements in regions 2 and 4 [27], [28]. Region 2, which is located in the first intron, contains binding sites for B-cell-specific transcription factors such as Pax5 and E [27], [28], [31], as well as E2F- and c-Myb-binding sites, which exert a strong silencing effect on the promoter [28]. Region 4, located about 8-kb upstream of the transcription-initiation site, contains stimulation-responsive elements for STAT6, NF-κB, Smad, and C/EBP. A previous *in vitro* reporter assay of mutations in these elements led us to propose a balanced regulation model of the *Aicda* promoter, in which B-cell-specific and stimulation-responsive enhancers coordinate to counteract the silencers, de-repressing the *Aicda* promoter [28].

To understand the physiological mechanisms regulating *Aicda*, here we used BAC DNA to generate several lines of transgenic mice, and used these mice to verify the *in vivo* activity of *Aicda* regulatory elements identified by the previous *in vitro* reporter assay [28]. The present study clearly demonstrates that *Aicda* is regulated *in vivo* by the balance between the positive and negative transcription factors that were identified in our *in vitro* experiments.

## Materials and Methods

### Ethics Statement

Animal housing and experiments were performed according to the Animal Experiment Guidelines Riken Center for Developmental Biology and the Regulation on Animal Experimentation at Kyoto University. All protocols involving mice were approved by the Kobe Animal Experiments Committee at RIKEN and the Animal Research Committee of the Graduate School of Medicine, Kyoto University (Permit Number: AH13-03-59 and 10055). Mice were euthanized according to the Animal Experiment Guidelines Riken Center for Developmental Biology and the Regulation on Animal Experimentation at Kyoto University before removing lymphoid organs.

### Generation of Transgenic Mice

**Figure 1:**
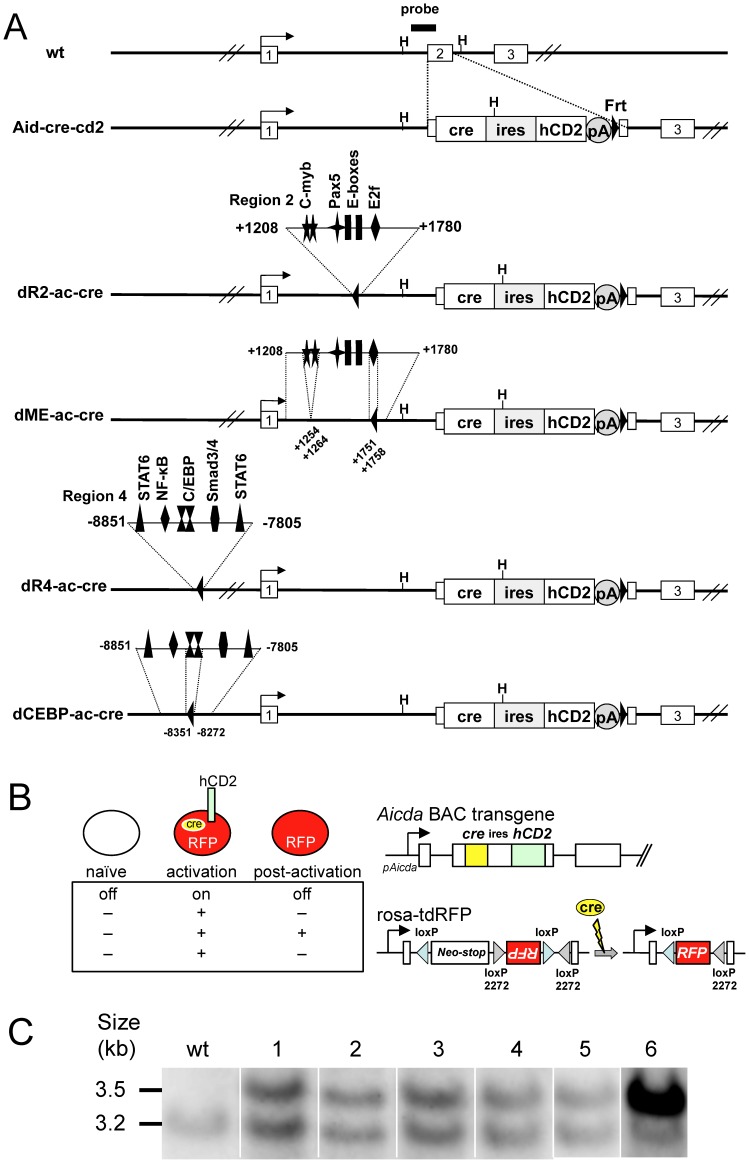
Generation of BAC transgenic mice. (A) *Aicda* on the BAC clone (RP24-68I7) was modified as described in Methods. Open boxes with numbers denote the first to third exons. An expression cassette for cre and human CD2 was inserted into the second exon as indicated. In the enlarged views of regulatory elements in regions 2 or 4 that were deleted or replaced by Frt sites (closed triangle), the number indicates the relative position (base pair) from the transcription initiation site. Binding elements are marked by a symbol showing the name of the factor. A thick line indicates the probe used for Southern blotting. The *Hind*III restriction sites adjacent to the probe are marked by an H. pA, poly A signal. (B) Characteristic cellular phenotype of Aid-cre-cd2 TG mice crossed with rosa-tdRFP mice. When stimulation induces the *Aicda* promoter (*pAicda*), the BAC transgene expresses cre recombinase and the hCD2 marker. Cre recombines loxP and loxP2227 sites in the indicator gene, rosa-tdRFP, and thereby irreversibly expresses RFP. RFP expression is maintained after *pAicda* activity declines. (C) Southern blot analysis with the probe defined in A: Genomic DNA from wild-type (lane wt), Aid-cre-cd2 (lane 1), dR2-ac-cre (lane 2), dME-ac-cre (lane 3), dR4-ac-cre (lane 4), dCEBP-ac-cre (lane 5), and Aicda-cre (lane 6) [17] mice was digested by *Hind*III; 3.2- and 3.5-kb bands correspond to endogenous and transgenic loci, respectively.

To generate BAC transgenic mice, we obtained the BAC clone RP24-68I7, which is 190 kb in length and harbors the entire *Aicda* gene, from BACPAC CHORI (http://bacpac.chori.org/) [32]. To generate the Aid-cre-cd2 construct, an expression cassette of cre IRES hCD2, the intracellular domain of which was deleted, was inserted into exon 2 of mouse *Aicda* on the BAC by homologous recombination in bacteria, as described previously [32] (Fig. 1A). We derived transgenic constructs with a series of deletions (Fig. 1A) from the Aid-cre-cd2 construct. The Red/ET recombination kit (Gene Bridges, Germany) was used according to the manufacturer’s instructions for all the BAC modifications. In brief, we constructed vectors carrying two homology arms (either an 80-bp synthesized oligonucleotide or a PCR product of approximately 0.5 kb) adjacent to the DNA region we wished to delete or replace. The homology arms were ligated with donor DNA containing PGK-Neo cassettes floxed by Frt sequences. DNA fragments for targeting were prepared by PCR and transfected into BAC-bearing E. coli with the pRedET plasmid. Recombinants were selected with kanamycin, and the PGK-Neo cassette was removed by transfecting the FLPe plasmid. When introducing secondary modifications to the Aid-cre-cd2 BAC construct, the Frt orientation was reversed in the second targeting construct to avoid accidentally deleting regions between the initial Frt site and the newly introduced Frts. Clones with the reversion were excluded after transfection with the FLPe plasmid.

The following primers were used to amplify the homology arms:

For the dR2-ac-cre transgene,

5′arm-dR2-Fwd: 5′-ATATGCAGCTCATGATGAGCCA-3′,

5′arm-dR2-Rv: 5′-AGGGGTGAGGGAGAGTGGGAGG-3′,

3′arm-dR2-Fwd: 5′- GTGCTGGATGGAGGTGCCCAGT-3′,

and 3′arm-dR2-Rv: 5′- GTTATAATAATAAGCACAGGTA-3′.

For the dCEBP-ac-cre transgene,

5′arm-dCEBP-Fwd: 5′- GAGCGTAGAGGTCAGTGGACAA -3′,

5′arm-dCEBP-Rv: 5′- CAACTTCTGCATTCCCCGATTTT-3′,

3′arm-dCEBP-Fwd: 5′- AGCTGGTGTCCAAAAATAGTGA-3′,

and 3′arm-dCEBP-Rv: 5′- CTCTTACAGATGCCGTACACAT-3′.

For the dR4-ac-cre transgene,

5′ homology arm (80 bp): 5′-TCACCACACTCAGTTTGAAGCCTTTTAATTTCATAGTATTCCATTTGAAAATTCTTTTTCTCACTATAGGGCTCGAGGAA-3′, and

3′ homology arm (80 bp):

5′-CCACACCCTCTCTTACAGATGCCGTACACATGCCATAGGAACAAATACAAAACCCTTGAGAATTCGATATACGAAGTTAT-3′.

The dME-ac-cre transgene was generated in two steps. First, we amplified the fragment containing the c-Myb-binding motifs using the primers 5′arm-ME-Fwd: 5′-CTTTTCCTTCTCCTCCTTCTCC-3′ and 5′arm-ME-Rv: CCTCTCCTCGGGATGTCCCTCC. We then cloned the amplified fragment into the pGEM-T easy vector (Promega). The whole plasmid was PCR-amplified with the primers myb-mut-F 5′-NNGGATCCACATCCTGAGCCCTCAAAAAGCA-3′ and myb-mut-R 5′-NNGGATCCTTGTCTAGCATGTGTGAGGTCTTC-3′. The PCR product was digested by *Bam*HI and self-ligated. The other homology arm, the adjacent region of the E2F site, was amplified with the primers 3′arm-ME-Fwd: 5′-GCTCCTAGCTAGAGTTGAGGGG-3′ and 3′arm-ME-Rv: 5′-TCTAATCCAGCCAGTTTTAAAC-3′. Two homology arms were ligated with both ends of the PGK-Neo cassette, and were used for homologous recombination in E. coli harboring Aid-cre-cd2. The nucleotide sequences of the modified area in the final constructs were checked by sub-cloning and sequencing.

Transgenic mice were generated by injecting supercoiled BAC DNA into CD-1 or C57BL/6 fertilized eggs; this was performed by the Laboratory for Animal Resources and Genetic Engineering, RIKEN Center for Developmental Biology (CDB) (Aid-cre-cd2, Acc. No. CDB0481T; dCEBP-ac-cre, Acc. No. CDB0482T; dR4-ac-cre, Acc. No. CDB0483T; dR2-ac-cre, Acc. No. CDB0484T: http://www.cdb.riken.jp/arg/TG%20mutant%20mice%20list.html) and the Reproductive Engineering Team at the Institute of Virus Research at Kyoto University. We used founder mice that had been backcrossed to C57BL/6 mice for more than five generations. All mice were bred and maintained in specific pathogen-free conditions at Kyoto University and at the RIKEN CDB.

Rosa-tdRFP [33] mice were bred and maintained in specific pathogen-free conditions at the Institute of Laboratory Animals at the Graduate School of Medicine, Kyoto University. These animals were intercrossed to obtain double-mutant animals.

### Immunization of Mice

Sheep red blood cells (SRBC) were washed by PBS for three times and were suspended in PBS at a concentration of 5×10^9^/ml. Hundred micro-l of the SRBC suspension was given intraperitoneally.

### Southern Blot and PCR Analysis

DNA for PCR and Southern blot analysis was obtained by tail biopsy. For Southern blotting, the DNA was digested with *Hind*III, separated on a 0.8% agarose gel, and transferred to a nylon membrane (Roche). The blots were air-dried, and the DNA crosslinked using UV light. The DIG-labeled DNA probes indicated in Fig. 1 were used for hybridization in ULTRAhyb Buffer (Ambion; Applied Biosystems). All procedures using the DIG application system (Roche) were performed according to the manufacturer’s recommendations. Screening of the transgenes was done by PCR with primers CreFP33 5′- CGGCATGGTGCAAGTTGAATAAC-3′ and CreRP34 5′- GCTAACCAGCGTTTTCGTTCTGC-3′, which amplify 458 bp DNA of the cre-coding region.

The following PCR primers were used to confirm the generated BAC constructs:

dR2-Fwd 5′-ATATGCAGCTCATGATGAGCCA-3′, dR2-Rv 5′- GTTATAATAATAAGCACAGGTA-3′; dR4-Fwd 5′-TGAATTGGTTCACTCCCCCTA-3′, and dR4-Rv 5′-CAGCTTACAGGAAACTTCCCA-3′.

### Antibody Staining, hCD2 Detection, and Cell Sorting

Single-cell suspensions from tissues, in FACS buffer (PBS supplemented with 4% FBS, 1 mM HEPES, and 0.6% sodium citrate), were stained with the following monoclonal antibodies conjugated with FITC, PE, PE-Cy7, APC, APC-Cy7, Biotin, or Pacific-Blue: antibodies specific for B220 (RA3-6B2), CD93 (AA4.1), IgM (R6-60.2), IgD (11–26), CD38 (90), CD3 (500A2), CD4 (GK1.5), CD8 (53-6.7), CD44 (IM7), CD62L (MEL-14), and hCD2 (RPA-2.10) (BD Biosciences or eBioscience). Biotinylated reagents were detected with either streptavidin-APC-Cy7 or streptavidin-APC (BD Biosciences). Stained cells were analyzed by FACSCantoII (BD Biosciences) or sorted by FACSAria. The acquired data were analyzed using FlowJo software (Tree Star, Inc.). Live lymphocyte populations were gated according to forward and side scatters, and 7-amino-actinomycin D staining.

### Quantitative Real-time PCR

Total RNA was extracted by TRIzol reagent (Invitrogen Life Technologies), and cDNA was synthesized with TaqMan® Reverse Transcription Reagents (Applied Biosystems). Gene expression was assessed by real-time PCR using iQ Sybr green supermix (Bio-Rad Laboratories, Inc.). The data were normalized to the *Gapdh* expression. The following primers were used: *hCD2*-forward 5′-GACCACCAGCCTGAGTGCAA-3′, *hCD2*-reverse 5′-GCTCCTCATCATTTCTCCGAC-3′; *Gapdh*-forward 5′-TGTGTCCGTCGTGGATCTGA-3′, *Gapdh*-reverse 5′-CCTGCTTCACCACCTTCTTGAT-3′.

## Results

### Construction of *Aicda* BAC Transgenic Mice

To examine the *in vivo* function of elements that regulate *Aicda*, we used a BAC transgenic strategy. We used the BAC clone RP24-68I7, which covers the 190-kb region of *Aicda* and its flanking regions, to establish a reliable system for monitoring *Aicda* expression [17]. We used a basic construct, Aid-cre-cd2, which harbors a bicistronic expression cassette for human CD2 and cre in the AID coding region (Fig. 1A) [32]. The *Aicda* BAC contains three regulatory regions (regions 2, 3, and 4) in addition to the promoter (region 1), as assessed by an *in vitro* culture system [16], [27], [28]. The locations and binding motifs of regions 2 and 4 are described above (Fig. 1A and the reference [28] for detailed information).

To examine the *in vivo* function of the *Aicda* regulatory elements identified *in vitro*, we added deletion mutations to the basic construct to obtain four additional constructs: dR2-ac-cre, in which region 2 was entirely deleted; dME-ac-cre, in which the region 2 c-Myb- and E2F-binding sites–the silencing elements–were deleted; dR2-ac-cre, in which region 4 was entirely deleted; and dCEBP-ac-cre, which contains a restrictive deletion of the two C/EBP sites (Fig. 1A and Fig. S1). We generated transgenic mice carrying these BAC constructs and crossed them with the rosa-tdRFP reporter mouse [33]. In this system, *Aicda* promoter activation can be visualized by hCD2 and RFP for ongoing and past AID expression, respectively (Fig. 1B). It was previously reported, that the cre-loxP-mediated irreversible expression of reporters at the rosa26 locus enables more sensitive detection than the direct staining of hCD2, even allowing the visualization of transient and/or marginal activation of the *Aicda* promoter in immature B cells and memory T cells [17]. All mice described in this paper were crossed with the rosa26-tdRFP reporter strain unless otherwise described.

### *Aicda* Expression is Positively Regulated by Regions 2 and 4

**Figure 2:**
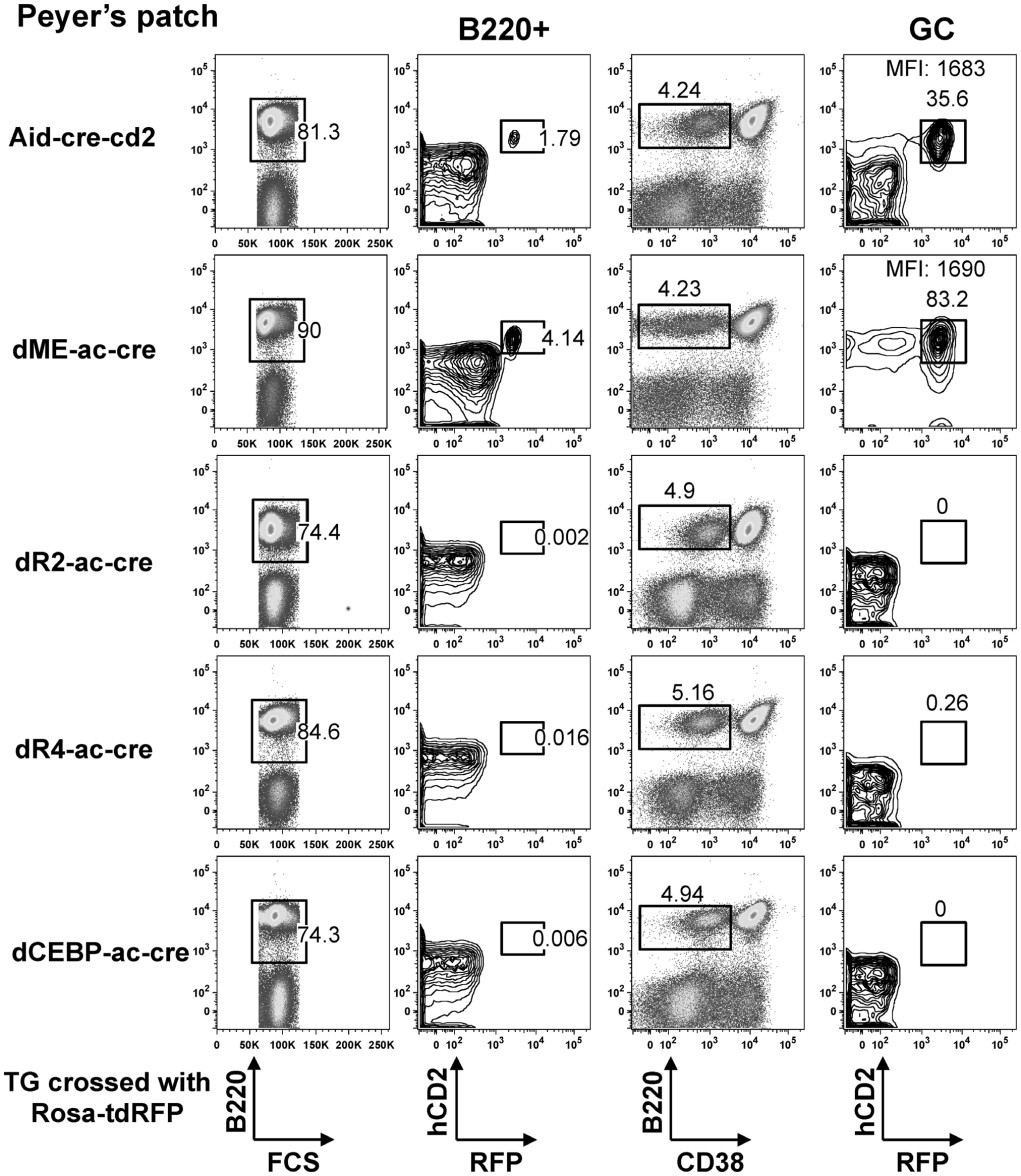
Dual-reporter hCD2 and RFP expressed in *in vivo*-activated B cells of each strain of rosa-tdRFP-crossed BAC transgenic mice. RFP and hCD2 in B cells (B220+) or germinal-center (GC) B cells (B220+CD38–) in Peyer’s Patches (mice at 8 weeks of age) from Aid-cre-cd2 line 1; dME-ac-cre line 1; dR2-ac-cre line 1; dR4-ac-cre line 1; or dCEBP-ac-cre line 1 mice. All mice were crossed with the rosa-tdRFP reporter strain. The mean fluorescent intensity (MFI) of hCD2 staining of the gated population and the percentage within the gate are indicated. FACS results are representative of three independent experiments with three mice each.

We chose 3 or 5 lines of transgenic mice for each construct (Table 1), each line carrying a single copy of the transgene (approximately 4 transgene copies were detected in the transgenic mouse described previously) (Fig. 1C) [17]. In all of these Aid-cre-cd2 lines, about one percent of the splenic B cells were RFP+, indicating that the basic BAC transgene was expressed similarly in these animals (Table 1). RFP and hCD2 were strongly expressed in germinal-center B cells, in which *Aicda* is actively transcribed; about 35% of the B220+CD38–gated population was positive for both reporters (Fig. 2). We next examined germinal-center B cells in the Peyer’s patches of mice carrying manipulated BAC reporter constructs. Deletions of region 2 or 4 completely abolished the *Aicda* expression on the transgene (Fig. 2). *Aicda* was similarly inactivated in the independent founder lines dR2-ac-cre and dR4-ac-cre (five each) (Table 1). We conclude that both region 2 and region 4 are essential for normal *Aicda* expression in activated B cells *in vivo*.

**Table 1:** Percentage of RFP+ Lymphocytes (mean ± SEM).

		Spleen		mLN		Peyer’s patch	
Construct	line	B(%)	T(%)	B(%)	T(%)	B(%)	T(%)
**Aid-cre-cd2**	#1	1.0±0.05	0	0.9±0.2	0	2.8±0.3	0
	#2	1.1±0.2	0	0.8±0.2	0	2.9±0.7	0
	#3	1.3±0.4	0	0.8±0.1	0	3.1±0.2	0.03±0.02
**dR2-ac-cre**	#1	0	0	0	0	0	0
	#2	0	0	0	0	0	0
	#3	0	0	0	0	0	0
	#4	0	0	0	0	0	0
	#5	0	0	0	0	0	0
**dR4-ac-cre**	#1	0	0	0	0	0.005±0.001	0
	#2	0	0	0	0	0	0
	#3	0	0	0	0	0	0
	#4	0	0	0	0	0	0
	#5	0	0	0	0	0	0
**dCEBP-** **ac-cre**	#1	0	0	0	0	0	0
	#2	0	0	0	0	0	0
	#3	0	0	0	0	0	0
	#4	0	0	0	0	0	0
	-#5	0	0	0	0	0	0
**dME-ac-** **cre**	#1	1.9±0.1	0	1.2±0.2	0.01±0.01	4.2±0.8	0.06±0.01
	#2	1.9±0.2	0.07±0.06	1.2±0.1	0.02±0.01	4.4±0.8	0.06±0.06

All animals were crossed with rosa-tdRFP.

Three mice from each line were analyzed.

Percentage of RFP+ B cells in mice carrying the dME-ac-cre transgene was significantly higher than in mice carrying the Aid-cre-cd2 transgene. P = 0.0067 (Spleen), P = 0.0034 (mLN), P = 0.0021 (Peyer’s patch) by two-tailed unpaired Student’s t-test. mLN, mesenteric lymph node.

Region 4 contains binding motifs for NF-κB, STAT6, and Smad3/4, which are involved in the signaling pathways of CD40, IL-4, and TGF-β, respectively [34]–[36]; an *in vitro* reporter assay also demonstrated the importance of two C/EBP sites in this region [28]. The dCEBP-ac-cre construct, in which the two C/EBP sites in region 4 were deleted, along with the intervening sequence (Fig. S1), consistently failed to express the *Aicda* reporters, indicating that these C/EBP sites are in fact indispensable for region 4’s enhancer activity in activated germinal-center B cells (Fig. 2 and Table 1).

### E2F and c-Myb Motifs Suppress *Aicda* Activity

**Figure 3:**
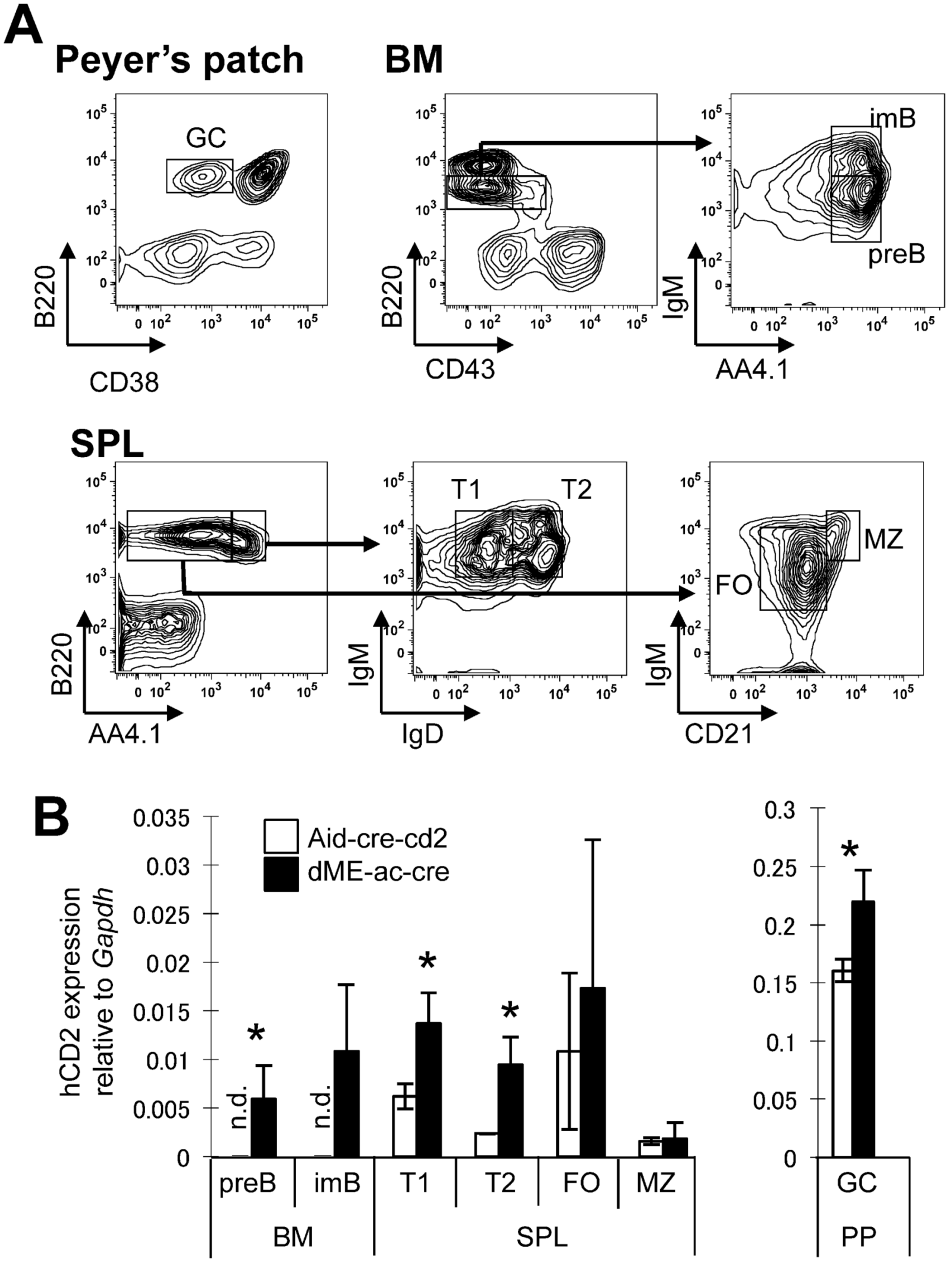
Positive correlation between hCD2 mRNA and the number of RFP+ cells among germinal-center B cells in dME-ac-cre mice. (A) Representative sorting and gating strategies for samples from the germinal centers of Peyeŕs patches (PP), bone marrow (BM), and spleen (SPL) of 8-week-old Aid-cre-RFP mice. Cells were stained by the indicated markers for FACS analysis. Sorting gates are indicated. (B) RT-qPCR analysis of hCD2 in sorted cells. Aid-cre-cd2 line 1 and dME-ac-cre line 1 mice (8 weeks old) were used; values were normalized to the *Gapdh* expression. Data represent means ± s.d. (bars) of three independent experiments. Asterisk indicates a mild statistical difference between Aid-cre-cd2 and dME-ac-cre mice, assessed by a two-tailed Student’s t-test (preB, *p* = 0.042; T1, *p = *0.02; T2, *p = *0.014; GC, *p = *0.024). T1, transitional B cell; T2, transitional B cell 2; MZ, marginal zone B cell; FO, follicular B cell; GC, germinal-center B cell; n.d., not detectable.

In contrast to the enhancer function of the C/EBP sites in region 4, the E2F- and c-Myb binding sites act as silencers *in vitro* [28]. To determine whether these binding sites were responsible for silencing *Aicda* in germinal-center B cells, we examined transgenic lines carrying the dME-ac-cre construct. In contrast to the dR2-ac-cre transgenic mice, both reporters were expressed at elevated levels in the Peyer’s patch germinal-center B cells in dME-ac-cre transgenic mice compared with Aid-cre-cd2 mice (Fig. 2). The percentage of germinal-center B cells that were positive for hCD2 or RFP in the dME-ac-cre mice was almost double that seen in Aid-cre-cd2 transgenic mice. The mean fluorescent intensity of hCD2+ B cells was similar in the Aid-cre-cd2 and dME-ac-cre mice (Fig. 2), suggesting that the efficiency of *Aicda* expression was similar once activated. In addition, the percentage of RFP-positive B cells in various lymphoid organs was significantly higher in the dME-ac-cre than in the Aid-cre-cd2 transgenic mice (Fig. 2 and Table 1). These results suggest that the *Aicda* promoter was more readily activated in dME-ac-cre than in Aid-cre-cd2 mice. We sorted germinal-center B cells, as well as B cells at other developmental stages, and used RT-qPCR to determine whether the increased percentage of cells positive for hCD2 or RFP correlated with the mRNA level. Consistent with the FACS analysis, the reporter-gene mRNA levels in the sorted cells were significantly higher in the dME-ac-cre than in the Aid-cre-cd2 transgenic mice (Fig. 3). Taken together, these results indicate that the E2F and c-Myb elements negatively modulate the *Aicda* promoter’s activity in germinal-center B cells *in vivo*.

**Figure 4:**
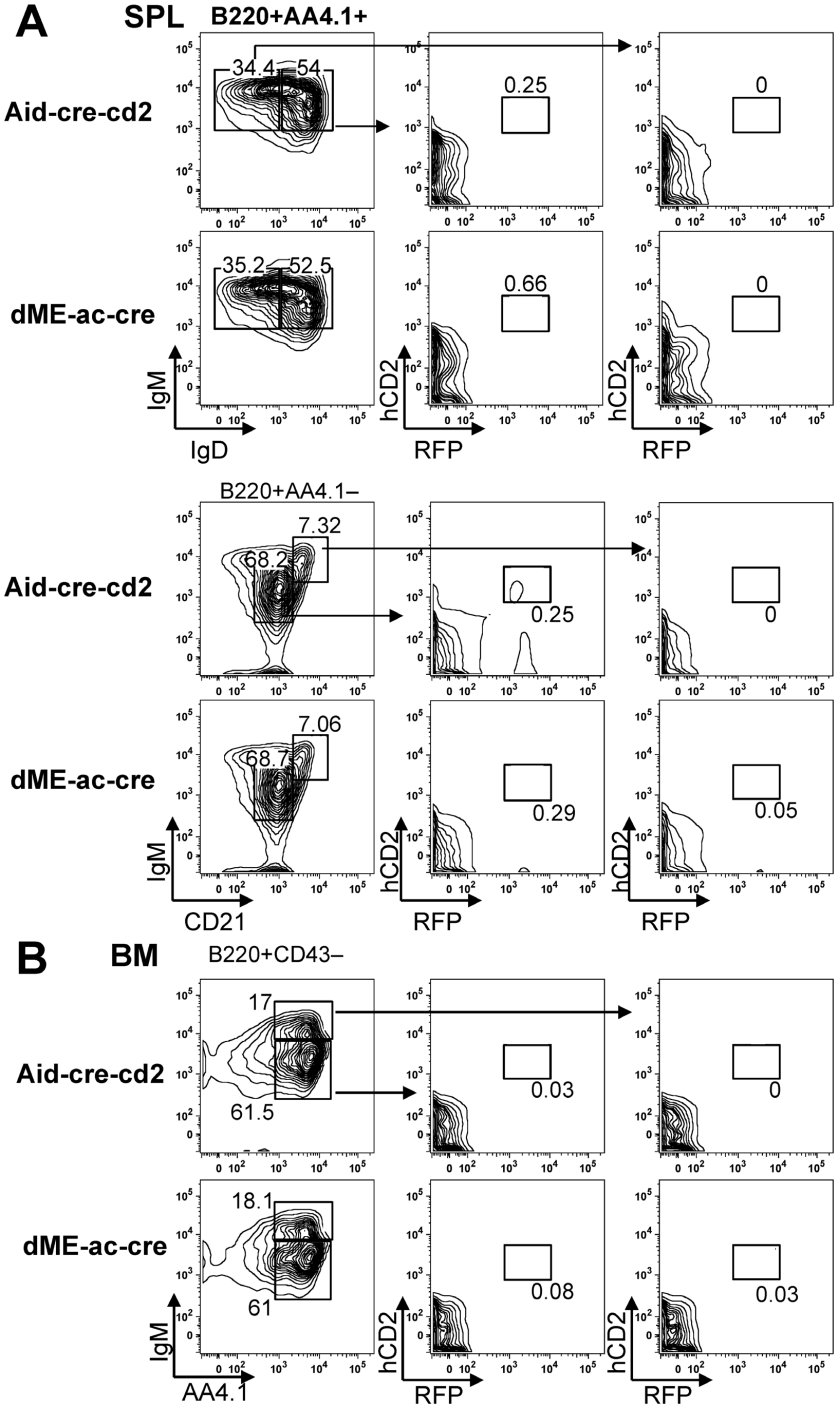
FACS analysis of hCD2 and RFP expression in Aid-cre-cd2 and dME-ac-cre B cell-lineage lymphocytes. (A) Spleen (SPL) and (B) bone marrow (BM) B cells of 8-week-old Aid-cre-cd2 line 1 and dME-ac-cre line 1 mice were stained for the indicated markers for FACS analysis. Numbers shown in each gate indicate the percentage of cells. FACS results are representative of three independent experiments with three mice each.

To determine whether deleting these silencer elements, E2F and c-Myb, would upregulate the modest AID expression found in immature B cells, we examined the reporter expression in developing B cells in dME-ac-cre mice. These B cells did not robustly express hCD2 or RFP in immature stages (Fig. 4AB). However, the hCD2 mRNA levels in the sorted preB and transitional B cells were elevated in the dME-ac-cre (*p*<0.05) as compared to the Aicda-cre-cd2 mice (Fig. 3B). Notably, the RT-qPCR detection appeared to be more sensitive than BAC reporters. These results support the idea that the E2F and c-Myb elements in region 2 negatively regulate the *Aicda* promoter in immature B cells.

**Figure 5:**
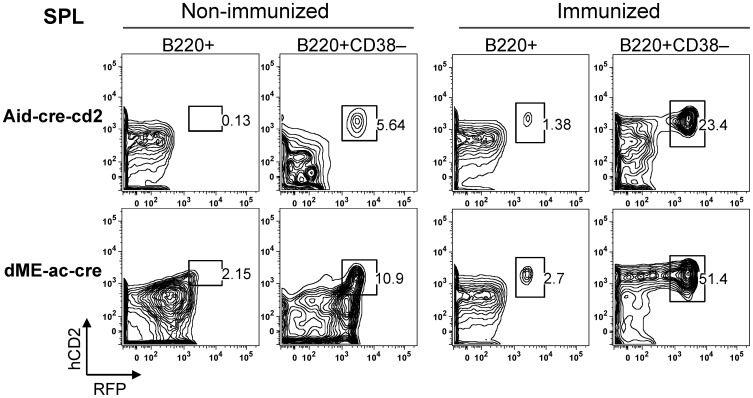
FACS analysis of hCD2 and RFP expression in immunized mice. Aid-cre-cd2 line 1 and dME-ac-cre line 1 mice (12 weeks of age) were immunized with SRBC and sacrificed on day 7 after immunization. The percentage of hCD2+ and RFP+ cells among B cells or germinal-center (GC) B cells is indicated. SPL, spleen. FACS results are representative of two independent experiments.

Germinal centers in Peyer’s patches develop spontaneously in response to continuous stimulation from gut flora [37]. To determine whether the increased induction of RFP-positive cells in the dME mutant was dependent on chronic stimuli, we immunized naïve mice by intra-peritoneal sheep red blood cell (SRBC) injection and analyzed the germinal-center B cells on day 7, the early phase of the immune response [38]. Germinal-center B cells in the spleen were gated as B220+CD38– cells [39]. As in the Peyer’s patches, a much higher percentage of dME-ac-cre spleen germinal-center B cells were positive for RFP or hCD2 than Aicda-cre-cd2 cells (Figure 5). Therefore, the c-Myb and E2F elements’ silencing activity negatively regulates *Aicda* promoter activation at the initiation stage of B-cell activation.

### Deletion of Silencer Elements in Region 2 does not Enhance the *Aicda* Expression in T cells

**Figure 6:**
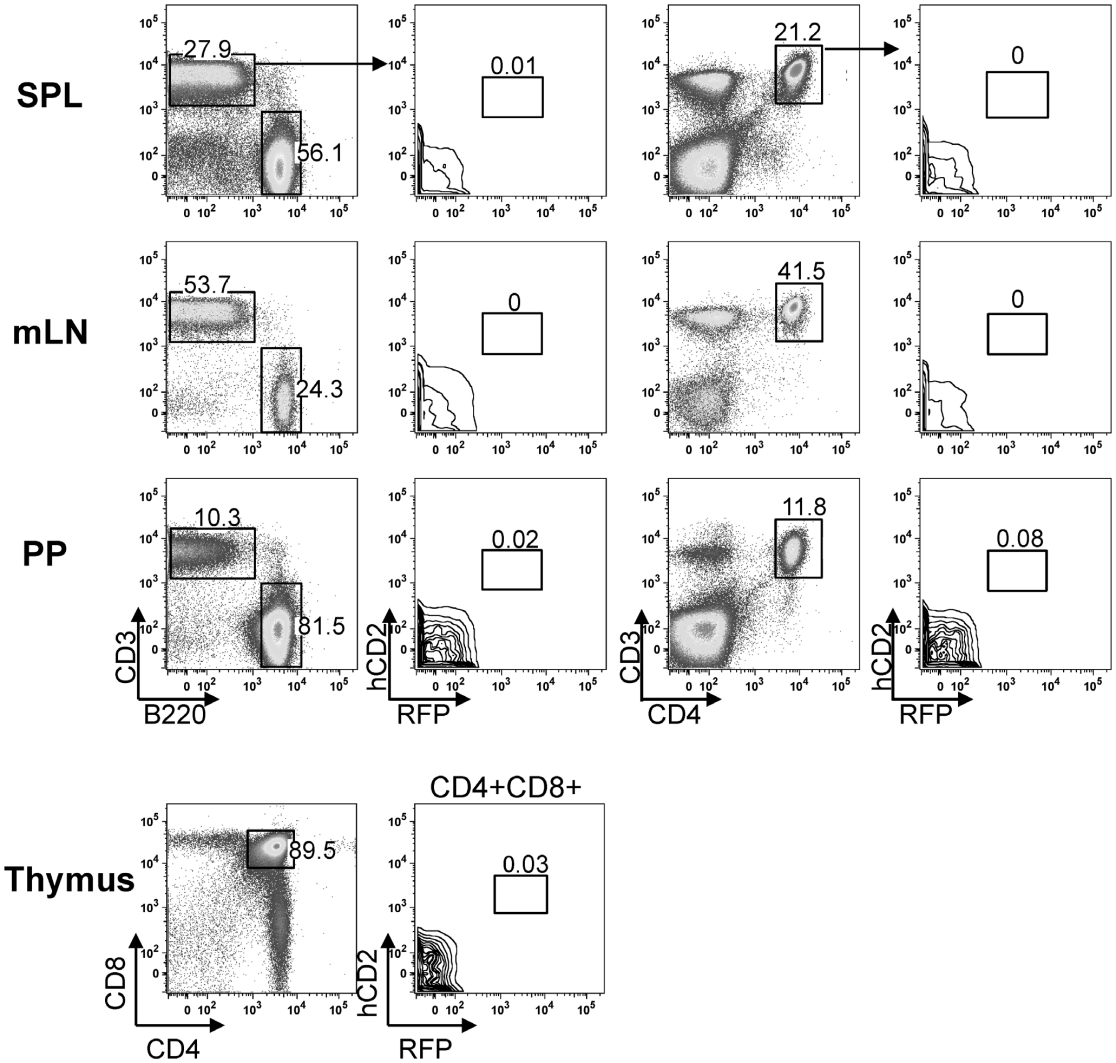
Lack of hCD2 and RFP expression in the peripheral T cells of dME-ac-cre mice. FACS analysis of hCD2 and RFP expression in naïve CD3+ or CD4+CD3+ T cells isolated from (A) spleen (SPL), (B) mesenteric lymph nodes (mLN), (C) Peyer’s patches (PP), and CD4+CD8+ T cells isolated from the thymus (D). Data are representative of three independent experiments. Data shown are from a 24-week-old mouse. Numbers in panels show the percentage of gated cells of each gate.

Since region 2’s silencing activity is not limited to B cells [28], [40], it might be responsible for repressing *Aicda* in other cell lineages. *Aicda* is weakly expressed in chronically activated T cells that coincide with the development of the IL-10-producing T-cell subpopulation [17]. Reporter expression did not indicate a significant fraction of RFP+ cells among the T cells of the dME-ac-cre mice (Fig. 6). Although we were able to detect RFP+ T cells in our previously reported transgenic mice, which carried multiple copies of the basic construct *Aid-cre-cd2,* this was not the case with the present single-copy transgenic mice (Table 1). Since the dME-ac-cre construct was efficiently expressed in germinal-center B cells, this lack of expression is not caused by the functional inactivation of the transgene itself. Although c-Myb and E2F strongly suppress the *Aicda* promoter, even a marginal level of *Aicda* expression in T cells appears to require additional positive regulation [28].

## Discussion

The AID molecule is central to the antigen-induced alteration of immunoglobulin genes in activated B cells, and its mutagenic activity can induce tumorigenesis in various types of cells [10]. AID is also thought to play a part in immature B-cell selection and T-cell activation, though it is expressed at much lower levels in these cells than in activated B cells [17], [25], [26]. It is thus clear that the *Aicda* promoter is finely regulated by cell lineage and the cellular environment. Determining how the *Aicda* gene is repressed in most cells, moderately expressed in response to some environmental stimuli, and fully expressed in activated B cells is critical for clarifying AID’s physiological and pathological functions. However, *Aicda* expression under physiological conditions has not been elucidated in previous *in vitro* or *ex vivo* cellular studies [16], [27]–[31], [41].

### Region 2’s Critical Roles in B-cell-specific AID Expression

In the present study, we demonstrated that regions 2 and 4 are important for inducing *Aicda* in activated B cells *in vivo*. Although we did not evaluate the activity of region 3 [16], all of the constructs used in this study contained region 3. Therefore, it is clear that region 3 cannot functionally compensate for the lack of region 2 or region 4. Region 2 contains binding sites for B-cell-specific transcription factors, namely, the Pax5 and E proteins. The present *in vivo* results are consistent with our previous *in vitro* report showing enhancer activity in this region [28], [31]. In addition to the B-cell-specific enhancers, E2F- and c-Myb-binding sites in region 2 functioned as silencers in the *in vitro* reporter assay. These silencers counteracted positive regulatory elements in region 2 and region 4, in both B and non-B cells [28]. To elucidate the mechanism for lineage-specific and activation-specific AID induction, it was important to evaluate the E2F and c-Myb sites’ silencing function under physiological conditions. As expected, these elements disturbed *Aicda* promoter activation in *in vivo-*activated B cells. Deletion of the E2F and c-Myb sites (dME-ac-cre) drastically increased the percentage of hCD2-positive B cells in germinal centers, suggesting that these elements reduce the frequency of *Aicda* promoter activation within the gated population. On the other hand, when comparing the Aid-cre-cd2 and dME-ac-cre hCD2-positive germinal-center B cells, the mean fluorescent intensity of hCD2 staining was not significantly different. Since the hCD2 fluorescent intensity correlates with the reporter gene’s transcription efficiency in individual cells, this observation indicates that the E2F and c-Myb sites do not affect the active reporter gene’s transcription efficiency. These results may suggest that E2F and c-Myb negatively regulate the *Aicda* promoter’s activation, but do not affect its transcription efficiency once activated.

*Aicda* is usually expressed strongly only in activated B cells. If *Aicda* silencing were not stably maintained in other cells, even minimal exposure to various environmental stimuli could induce this dangerous internal mutagen. In this context, E2F proteins are known to associate with the pRB protein to function as a constitutive and tentative gene repressor [42]. Because the silencers in region 2 act in other cell lineages in addition to B cells [28], these silencers would also be expected to contribute to B-cell-specific *Aicda* suppression. We expected to observe ectopically expressed *Aicda* reporters in the absence of the silencers (dME-ac-cre mice). However, we did not observe ectopic or enhanced reporter expression in either immature B cells or developing T cells, in which the *Aicda* promoter is activated modestly under normal conditions [17]. This finding suggests that removing these silencers is not sufficient to induce ectopic *Aicda* expression. It is likely that the activity of the B-cell-specific enhancer proteins (Pax5 and E proteins) is not sufficient to activate the *Aicda* promoter in most cells, including immature B cells and activated T cells; this indicates that a coordinated balance between enhancers and silencers is critical for lineage- and activation-specific AID expression. Alternatively, there might be other unidentified silencers that are required for maintaining lineage specificity. However, this is less likely, since previous extensive deletion analysis did not find other obvious silencer elements, either in the AID gene or in its flanking regions [28].

A previous study clearly showed that a BAC transgenic system using cre recombinase coupled with floxed RFP can detect low-level *Aicda* expression [17]. However, we could not visualize *Aicda* expression records in immature B and activated T cells using this system in the present study. Since *Aicda* expression in these cells has been confirmed by other methodologies by various groups, it is unlikely that our previous observation was an artifact [16], [17], [23], [24]. Rather, it seems that the sensitivity of the current study’s system is lower than that reported by Qin et al. (2011). It is likely that this difference is due to the number of transgene copies; transgenic mice used in the previous study carried about 4 copies, while the current study used only animals with single-copy integration.

### Region-4 Enhancers Positively Regulate *Aicda*

Region 4’s role in *Aicda* regulation was first demonstrated *in vitro* by Tran et al. [28]. Our current study convincingly demonstrated *in vivo* the critical role of region 4’s enhancer elements, which include binding sites for NF-κB, STAT6, Smad3/4, and C/EBP. NF-κB, STAT6, and Smad are major transcription factors in the CD40, IL-4, and TGF-β signaling pathways, respectively. Although dCEBP-ac-cre selectively deleted only the tandem C/EBP-binding elements, leaving the other elements intact, it completely abolished the expression of *Aicda* reporters. This demonstrated C/EBP’s indispensable role in regulating *Aicda*, as was previously shown *in vitro*.

While some factors are well-known to contribute to CSR induction, the involvement of C/EBP proteins has not been previously appreciated. Chromatin immunoprecipitation assays have suggested that C/EBPβ binds to region 4 [28]. Since C/EBPβ can physically interact with NF-κB and modulate its activity [43]–[45], C/EBPβ and NF-κB can function cooperatively and are often involved in signaling pathways mediated by toll-like receptors and cytokines, which are associated with inflammatory reactions [43], [45]–[48].

For example, in renal medullary interstitial cells, a mutation of the C/EBP-binding site abolishes NF-κB-dependent activation of the COX2 promoter, which contains a tandem array of NF-κB- and C/EBP-binding sites [45]. These results suggest that the C/EBP pathway dominantly regulates the tonicity-induced COX2 expression. The NF-κB- and C/EBP-binding sites in region 4 are separated by almost 100 bp, which is relatively close, although not as close as in the COX2 gene. It is well established that AID is induced by CD40 stimulation, which activates NF-κB. In this context, C/EBPβ’s critical role in *Aicda* expression *in vivo* is reasonable. It will be interesting to elucidate the molecular mechanisms by which C/EBP and NF-κB interact and cooperatively regulate *Aicda* expression. In summary, the activation of AID expression *in vivo* requires not only B-cell-specific enhancers in region 2, but also stimulation-dependent enhancers in region 4.

## Supporting Information

Figure S1 **Confirmation of deletion/replacement of regulatory elements on BAC constructs.** PCR analysis confirmed the deleted of region 2 (0.5-kb) in dR2-ac-cre and that of region 4 (0.8-kb) in dR4-ac-cre (A) (B). Sequencing analyses to confirm the modifications introduced to dME-ac-cre (C) and dCEBP -ac-cre (D) were shown. The wild type sequences around the targeted elements (boxed) are presented. Sequencing chromatograms were shown below; corresponding region were indicated by dotted lines.
(PDF)
