# Supplementary figures and images for: In Vivo Analysis of Aicda Gene Regulation: A Critical Balance between Upstream Enhancers and Intronic Silencers Governs Appropriate Expression
Source: PLoS One. 2013 Apr 16;8(4):e61433. doi: 10.1371/journal.pone.0061433 (PMC3628980; doi:10.1371/journal.pone.0061433)

Figure S1

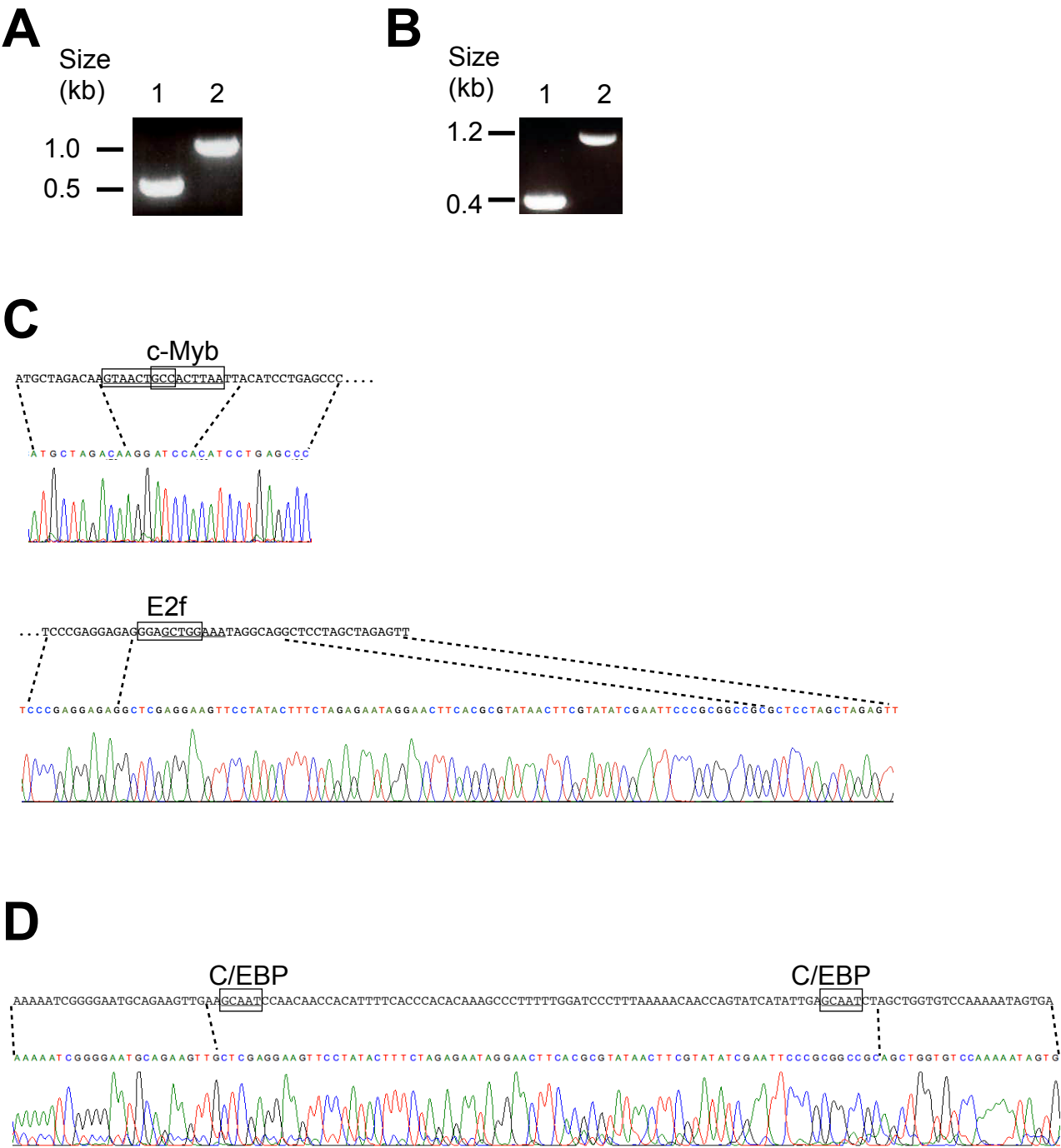

Supplement: Figure S1 — Confirmation of deletion/replacement of regulatory elements on BAC constructs. PCR analysis confirmed the deleted of region 2 (0.5-kb) in dR2-ac-cre and that of region 4 (0.8-kb) in dR4-ac-cre (A) (B). Sequencing analyses to confirm the modifications introduced to dME-ac-cre (C) and dCEBP -ac-cre (D) were shown. The wild type sequences around the targeted elements (boxed) are presented. Sequencing chromatograms were shown below; corresponding region were indicated by dotted lines. (PDF) [file pone.0061433.s001.pdf]
